# Comprehensive evaluation of resistance effects of pyramiding lines with different broad-spectrum resistance genes against *Magnaporthe oryzae* in rice (*Oryza sativa* L.)

**DOI:** 10.1186/s12284-019-0264-3

**Published:** 2019-03-01

**Authors:** Yunyu Wu, Ning Xiao, Yu Chen, Ling Yu, Cunhong Pan, Yuhong Li, Xiaoxiang Zhang, Niansheng Huang, Hongjuan Ji, Zhengyuan Dai, Xijun Chen, Aihong Li

**Affiliations:** 1 Lixiahe Agricultural Research Institute of Jiangsu Province, Yangzhou, 225009 China; 2 Jiangsu Collaborative Innovation Center for Modern Crop Production, Nanjing, 210095 China; 3 Jiangsu Key Laboratory of Crop Genomics and Molecular Breeding, Yangzhou University, Yangzhou, 225009 China; 4 Colleges of Horticulture and Plant Protection, Yangzhou University, Yangzhou, 225009 China

**Keywords:** Rice, Blast resistance, Broad-spectrum resistance, Polygene pyramiding line

## Abstract

**Background:**

Broad-spectrum resistance gene pyramiding helps the development of varieties with broad-spectrum and durable resistance to *M. oryzae*. However, detailed information about how these different sources of broad-spectrum resistance genes act together or what are the best combinations to achieve broad-spectrum and durable resistance is limited.

**Results:**

Here a set of fifteen different polygene pyramiding lines (PPLs) were constructed using marker-assisted selection (MAS). Using artificial inoculation assays at seedling and heading stage, combined with natural induction identification under multiple field environments, we evaluated systematically the resistance effects of different alleles of Piz locus (*Pigm*, *Pi40*, *Pi9*, *Pi2* and *Piz*) combined with *Pi1*, *Pi33* and *Pi54*, respectively, and the interaction effects between different *R* genes. The results showed that the seedling blast and panicle blast resistance levels of PPLs were significantly higher than that of monogenic lines. The main reason was that most of the gene combinations produced transgressive heterosis, and the transgressive heterosis for panicle blast resistance produced by most of PPLs was higher than that of seedling blast resistance. Different gene pyramiding with broad-spectrum *R* gene produced different interaction effects, among them, the overlapping effect (OE) between *R* genes could significantly improve the seedling blast resistance level of PPLs, while the panicle blast resistance of PPLs were remarkably correlated with OE and complementary effect (CE). In addition, we found that gene combinations, *Pigm*/*Pi1*, *Pigm*/*Pi54* and *Pigm*/*Pi33* displayed broad-spectrum resistance in artificial inoculation at seedling and heading stage, and displayed stable broad-spectrum resistance under different disease nursery. Besides, agronomic traits evaluation also showed PPLs with these three gene combinations were at par to the recurrent parent. Therefore, it would provide elite gene combination model and germplasms for rice blast resistance breeding program.

**Conclusions:**

The development of PPLs and interaction effect analysis in this study provides valuable theoretical foundation and innovative resources for breeding broad-spectrum and durable resistant varieties.

**Electronic supplementary material:**

The online version of this article (10.1186/s12284-019-0264-3) contains supplementary material, which is available to authorized users.

## Background

Rice blast caused by hemibiotropic fungal pathogen *Magnaporthe oryzae* is one of the most widespread and devastating rice diseases (Khush and Jena 2009). Due to its wide distribution and ability to survive under wide range of environmental conditions, yield loss caused by the rice blast fungus vary from 10% to 30%, meaning each year destroys abundant rice to feed more than 60 million people and economic losses over $70 billion dollars (Scheuermann et al. 2012; Skamnioti and Gurr 2009). Deployment of resistant cultivars by introducing resistance (*R*) genes into elite rice varieties were proved to be the most environmentally friendly and sustainable approach for blast control (Khush and Jena 2009). For the past decades, approximately 100 *R* genes and 350 quantitative trait loci (QTL) associated with blast resistance have been identified (Tanweer et al. 2015), of which 28 *R* genes have been cloned and functionally validated (Ashkani et al. 2016; Deng et al. 2017). However, most of these cloned and characterized *R* genes only confer resistance to one or a few isolates of *M. oryzae* follow the model of gene-for-gene interaction (Jia et al. 2000), and their resistance tend to retain an effective level for only a short time, especially when the varieties with *R* genes grown in large areas (Qu et al. 2006). Therefore, *R* genes showed broad-spectrum resistance to a number of isolates or races from one or different countries seem to be more reliable and sustainable in breeding programs (Skamnioti and Gurr 2009).

Many broad-spectrum *R* genes have been documented and validated, such as *Piz* (Kiyosawa 1967), *Pi1* (Yu et al. 1991), *Pi2* (Chen et al. 1996), *Pi9* (Liu et al. 2002), *Pi33* (Berruyer et al. 2003), *Pi54* (Sharma et al. 2005), *Pigm* (Deng et al. 2006) and *Pi40* (Jeung et al. 2007). *Piz* was originally reported in the U. S. cultivar Zenith and shown resistance to five U.S. races of blast (IH-1, IG-1, IC-17, IE-1 and IE1k) (RoyChowdhury et al. 2012). *Pi2* was firstly identified in a highly resistant *indica* rice cultivar 5173 (Zhou et al. 2006). Extensive field evalutaions indicated that *Pi2* showed resistance to 455 isolates collected from different regions of Philippines and most of the 792 isolates from 13 major rice regions of China (Chen et al. 1996). *Pi9* in the isogenic line 75–1-127 was origin from *Oryza minuta*, a tetraploid wild species of the *Oryza* genus and the lines carrying *Pi9* were highly resistant to 43 isolates collected from 13 countries (Qu et al. 2006). Genetic and mapping analysis also indicated *Pigm* and *Pi40* showed broad-spectrum resistance to several races of *M. oryzae* (Deng et al. 2006; Jeung et al. 2007). Literature reports indicated that these five resistance genes were different *R* gene alleles of the *Piz* locus located on the short arm near the centromere of rice chromosome 6 (Deng et al. 2006; Hayashi et al. 2004; Liu et al. 2002), and showed significant differences in patterns of resistance under different background (Wu et al. 2016, 2017). In addition, *Pi1* was originally identified on long arm of chromosome 11 in cultivar LAC23 (Mackill and Bonman 1992) and was proved to confer resistance to most of the 792 isolates from 13 major rice regions of China (Chen et al. 2001). *Pi54* gene was identified in a highly resistant cultivar Tetep and was mapped near *Pi1* locus, it was furtherly conferred broad-spectrum resistance against predominant races of *M. oryzae* in India (Sharma et al. 2010). Lastly, *Pi33* located on the short arm of chromosome 8, showed resistance to > 2000 isolates originating from 55 countries (Berruyer et al. 2003). MAS and conventional breeding together have facilitated the mentioned above broad-spectrum *R* genes to be incorporated in elite rice varieties to improve their blast resistance and durability (Deepti et al. 2017), especially *Pigm*, *Pi2* and *Pi9* at Piz locus to overcome blast diseases in rice has been successfully demonstrated (Jiang et al. 2015; Luo et al. 2017). However, due to high variability and emergence of new virulent races in the *M. oryzae* population, *R* genes such as *Pi9*, *Pi5* and *Pi3*(t) may loss broad-spectrum resistance to the pathogen populations when deployed individually (Variar et al. 2009).

Broad-spectrum *R* gene pyramiding helps the development of varieties with broad-spectrum and durable resistance to *M. oryzae* (Ellur et al. 2016; Gouda et al. 2013). However, which *R* gene pyramiding patterns show broad-spectrum and stable blast resistance is still little known. The resistance effects of PPLs with different broad-spectrum resistance genes, such as *Pi2*/*Pi1*, *Piz-t*/*Pi54* and *Pi1*/*Pi54* could be significantly improved as compared to the monogenic lines with single *R* gene (Jiang et al. 2012; Khan et al. 2018; Xiao et al. 2017). However, gene pyramiding does not always mean that the resistance spectrum could be improved. For example, the resistance level of PPL^*Piz5/Pita*^ was even lower than that of monogenic lines with *Piz5* (Hittalmani et al. 2000). Similarly, after pyramiding of *Pi9* with *Pi54*, the resistance level of PPL^*Pi9/Pi54*^ was also lower than that of monogenic lines with *Pi9* (Xiao et al. 2017). Thus, the combination patterns of *R* genes in rice varieties could affect the resistance level (Wu et al., 2015). Therefore, understanding the interaction effect between different broad-spectrum *R* genes, discovering *R* gene combinations with broad-spectrum and stable resistance is undoubtedly great significance for improvement of resistance to rice blast in breeding practice.

In previous study, the near-isogenic lines (NILs) of five resistance alleles of the Piz locus (*Pigm*, *Pi9*, *Pi40*, *Pi2* and *Piz*), and *Pi1*, *Pi33* and *Pi54* from other chromosome were constructed under Yangdao 6 (YD6) genetic background (Wu et al. 2016). In the present study, we crossed these lines to produce a total of fifteen PPLs, containing all possible gene combinations within a homogeneous genetic background. A large number of isolates collected from different ecological regions were used for seedling blast and panicle blast identification though artificial inoculation assays, combined with the natural induction under multiple field environments, the resistance effects of fifteen PPLs with different broad-spectrum *R* genes were evaluated, and the interaction effects between different *R* genes were analyzed to screen the best gene combinations with broad-spectrum and stable resistance. In addition, the basic agronomic traits of PPLs were investigated to evaluate the influence with different gene combination. These results will provide valuable theoretical foundation and new resistant germplasm for breeding broad-spectrum and durable resistant varieties.

## Results

### Development of PPLs in the genetic background of YD6

**Fig. 1:**
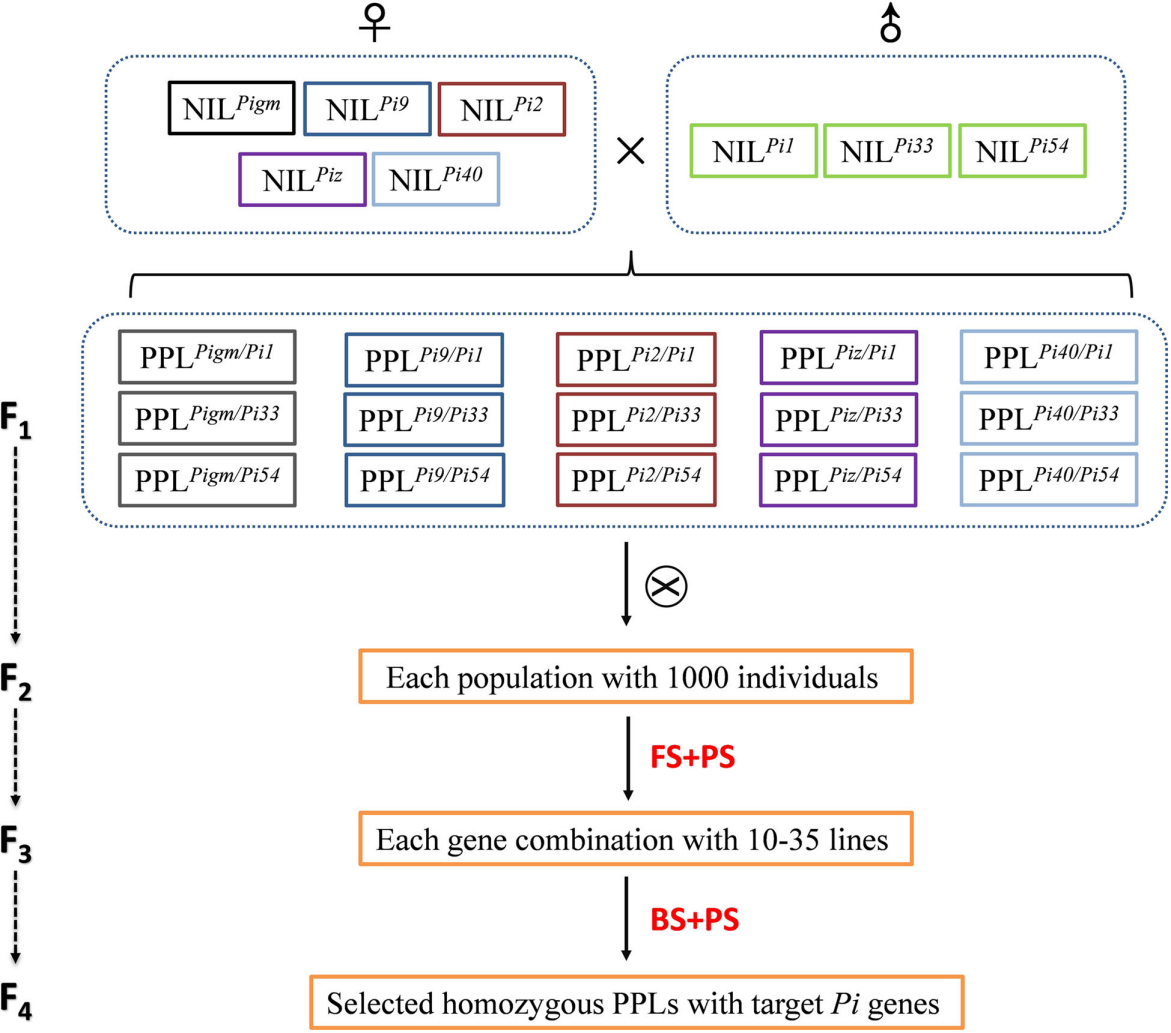
Breeding scheme for generation of PPLs. FS, foreground selection of Pi genes; BS, background selection by GBS analysis; PS, phenotype selection for yield and morphology related traits

Two set of NILs which harbored different broad-spectrum resistance genes were used for the development of PPLs in this study. The first set was composed of NILs with five alleles of Piz locus (*Pigm*, *Pi40*, *Pi9*, *Pi2* and *Piz*) on chromosomes 6 with YD6 as genetic background (Wu et al. 2016). The second set consisted of three NILs with YD6 as genetic background carried the broad-spectrum resistance gene *Pi1*, *Pi33* and *Pi54*, respectively. The 15 F_1_ combinations were developed by the way of genetic mating design of North Carollina II (NCII) using the first set of five NILs as the male parent and the second set of three NILs as female parent (Fig. 1). Then, these 20 plants from each 15 F_1_ combinations were screened for their heterozygosity with the help of gene specific markers and the “true” intercross. After selfed, the seeds of each combination were harvested and plant 1000 F_2_ populations, respectively. Foreground analysis of these populations with the gene specific markers and phenotype selection revealed that a total of 10 to 35 homozygous double gene positive plants were identified, respectively. Then, 2 to 4 homozygous F_3_ lines of each gene combination with agronomic traits similar to those of YD6 were selected for detection of the back ratio of genetic background using the genotyping by sequencing (GBS) method. Lines with the highest back ratio of genetic background were selected for subsequent resistance and agronomic trait evaluation. Sequencing results showed that the back ratios of genetic background of the target PPLs were more than 98.02%, ranging from 98.02% (PPL^*Pi2/Pi54*^) to 98.98% (PPL^*Pi2/Pi33*^) (Additional file 1: Table S1), indicated that the genetic background of all PPLs were almost fully identical to that of the recurrent parent YD6.

### Transgressive heterosis improve the seedling and panicle resistance spectrum of PPLs

**Fig. 2:**
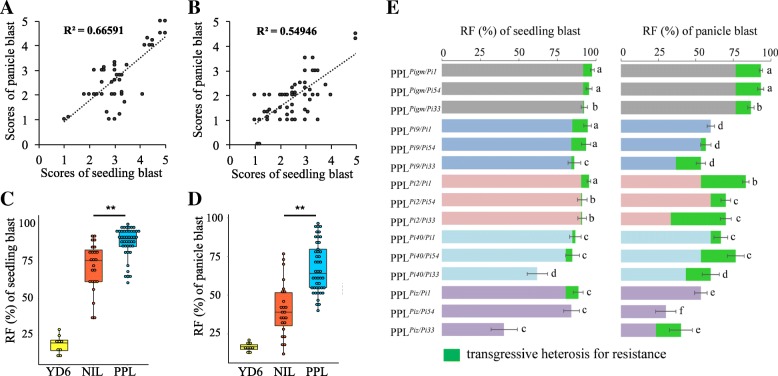
Resistance performances of PPLs for seedling and panicle blast resistance. **a** Correlation analysis of seedling blast resistance with panicle blast resistance of NILs, **b** Correlation analysis of seedling blast resistance with panicle blast resistance of PPLs, **c** Comprehensive comparative analysis on seedling blast RF of PPLs, NILs and the recurrent parent, **d** Comprehensive comparative analysis on panicle blast RF of PPLs, NILs and the recurrent parent, **e** transgressive heterosis for seedling blast and panicle blast resistance produced by PPLs after different broad-spectrum *R* gene pyramided

Eight NILs, fifteen PPLs and the recurrent parent were artificially inoculated with the collected isolates of *M. oryzae* at the seedling stage and heading stage respectively, and the results showed that there was a strong correlation between seedling blast and panicle blast resistance in NILs and PPLs, and the determination coefficient (R^2^) was 0.6659 and 0.5494, respectively (Fig. 2 a and b). We further analyzed the resistance level between PPLs and the NILs, and found that the seedling blast and panicle blast resistance levels of PPLs were significantly higher than that of NILs after pyramiding of different *R* genes (Fig. 2c, d). With regard to the seedling blast resistance, different gene combination produced different resistance effects, most of the fifteen gene combinations obtained transgressive heterosis (TH) except for three gene combinations of *Pi40*/*Pi33*, *Piz*/*Pi54* and *Piz*/*Pi33*. Despite the RF of NIL with *Pigm* was as high as 91.77%, it produced 5.90%, 3.80% and 0.64% of TH at the seedling stages when *Pigm* combined with *Pi1*, *Pi54* and *Pi33*, respectively, resulting in further improvement of resistance after gene combination (Fig. 2e). Similarly, there were twelve gene combinations obtained TH for panicle blast resistance except for three gene combinations of *Pi9*/*Pi1*, *Piz*/*Pi1* and *Piz*/*Pi54* at the panicle stage. Although the RF of NIL with *Pigm* was 76.67%, it produced 16.67%, 16.67% and 10.00% of TH when *Pigm* combined with *Pi1*, *Pi54* and *Pi33*, resulting in the RF of PPL^*Pigm/Pi1*^, PPL^*Pigm/Pi54*^ and PPL^*Pigm/Pi33*^ was as high as 93.33%, 93.33% and 86.67%, respectively. Interestingly, the RF of NIL with *Pi2* was only 33.33%, but the RF PPL^*Pi2/Pi1*^ and PPL^*Pi2/Pi33*^ was as high as 83.33% and 70.00% after *Pi2* combined with *Pi1* and *Pi33*, which produced 30.00% and 36.67% of TH, respectively (Fig. 2e). The PPLs present a broader resistant spectrum than NILs is that the PPLs’ partial resistant spectrum is overlapped with the resistant spectrum of the resistant *R* genes, PPL and NIL is of the same R gene that shows resistance to the same physiological isolates (Additional file 2: Figure S1). Furthermore, we also found that the TH for panicle blast resistance produced by most of PPLs is higher than that of seedling blast resistance. The mean value of TH for seedling blast resistance produced by PPLs was only 4.38%, and ranged from 0.63% to 9.91%. Correspondingly, the mean value of TH for panicle blast resistance was 16.94%, and distributed between 3.34% and 36.67%. Therefore, although the TH for seedling blast and panicle blast resistance was different produced by different PPLs with different broad-spectrum resistance genes combined, the broad-spectrum *R* gene pyramiding was still effective in broadening resistance spectrum and improving the durable resistance of the target materials.

### Interaction effects between different broad-spectrum *R* genes affect the resistance level of PPLs

**Fig. 3:**
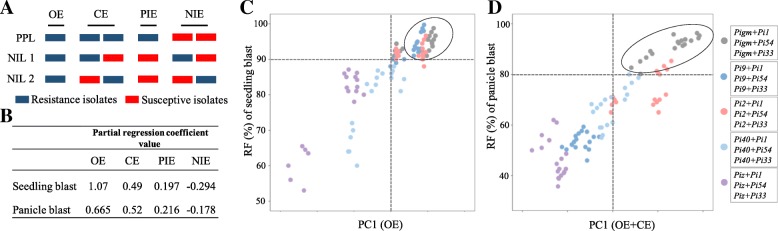
Different interaction effects in PPLs and their relationship with seedling blast and panicle blast RF of PPLs. **a** Four interaction effects produced in PPLs, **b** Correlation between interaction effects and RF of PPLs. β is the partial regression coefficient value of the linear regression, **c** Principal component analysis of OE with seedling blast RF of PPLs, **d** Principal component analysis of OE + CE with panicle blast RF of PPLs

**Fig. 4:**
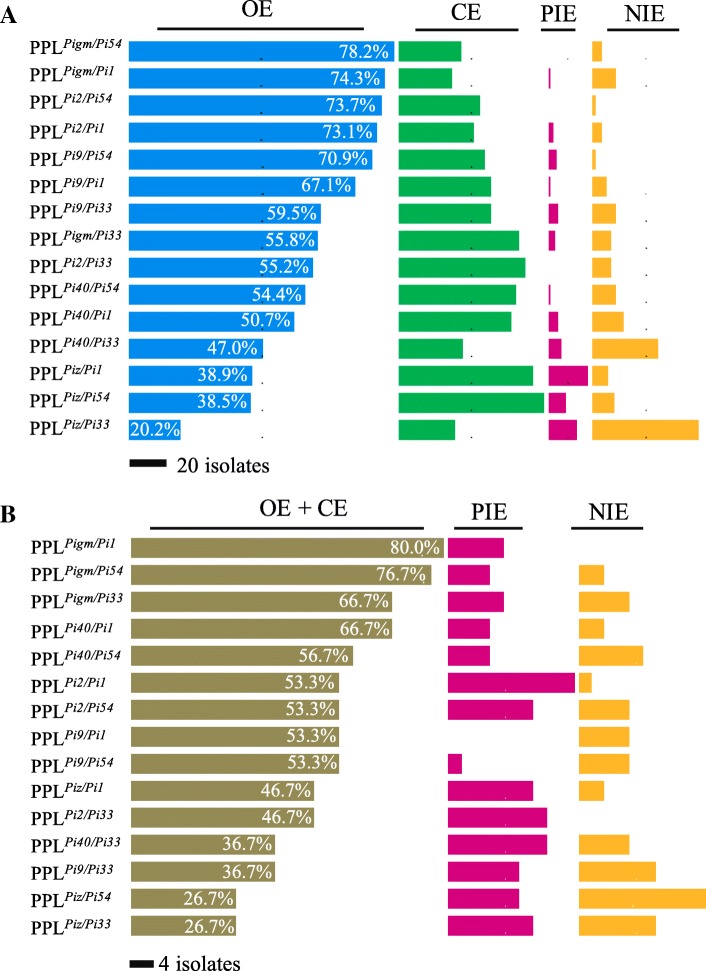
Four interaction effects affect the seedling and panicle blast resistance level of PPLs. **a** The interaction effects affect seedling blast resistance level of PPLs, **b** The interaction effects affect panicle blast resistance level of PPLs

In order to analyze the reason that PPLs’ RF is higher than NILs’ RF, we classified the resistance effect of PPLs against isolates of *M. oryzae* into four interaction effects, including: (1) PPLs’ partial resistant spectrum is overlapped with the resistant spectrum of both *R* genes, which is called the overlapping effect (OE); (2) PPLs’ partial resistant spectrum is overlapped with the resistant spectrum of the resistant *R* genes, which is called the complementary effect (CE); (3) PPLs present resistance to the physiological races that are sensitivity character to NILs with *R* gene, which is called the positive interaction effect (PIE); (4) PPLs present sensitivity to the physiological races that are resistance to NILs with *R* gene, which is called the negative interaction effect (NIE) (Fig. 3a). Analysis the relationship of the above four effects with RF of PPLs suggested that seedling blast RF of PPLs were mainly determined by OE (β =1.07), while the panicle blast RF of PPLs were remarkably determined by OE (β =0.665) and CE (β =0.52) (Fig. 3b). Principal component analysis was conducted to ascertain which effects are the major contributing factor in the RF of PPLs. The result also showed that OE as a major factor affected the seedling blast resistance (R^2^ = 65.21%), the RF of PPL^*Pigm/Pi1*^, PPL^*Pigm/Pi54*^, PPL^*Pigm/Pi33*^, PPL^*Pi9/Pi1*^, PPL^*Pi9/Pi54*^, PPL^*Pi2/Pi1*^, PPL^*Pi2/Pi54*^ and PPL^*Pi2/Pi33*^ were higher than 90% (Fig. 3c), and all of these nine PPLs had high OE, which was 74.3%, 78.2%, 58.8%, 67.1%, 70.9%, 73.1%, 75.6% and 55.19%, respectively (Fig. 4a). PPL^*Pi40/Pi33*^ and PPL^*Piz/Pi33*^ had the lowest RF and only displayed OE values of 39.24% and 15.19%, respectively. Different from the seedling blast resistance, the panicle blast resistance was positively correlated with OE and CE (R^2^ = 66.09%) (Fig. 3d). The RF of PPL^*Pigm/Pi1*^, PPL^*Pigm/Pi54*^, PPL^*Pigm/Pi33*^ and PPL^*Pi2/Pi1*^ were all higher than 80%, and their corresponding OE combined with CE also had high values, which were 80.0%, 76.7%, 66.7% and 53.3%, respectively, while PPL^*Piz/Pi54*^ had the lowest panicle blast RF and the value Of OE combined with CE was only 26.67%. The above results suggested that gene pyramiding of different broad-spectrum *R* gene produced different interaction effects and the interaction effect between different broad-spectrum *R* genes affect the resistance level of PPLs. Therefore, choosing the elite gene combination is key step to breed broad-spectrum resistance varieties in breeding practices.

### The PPLs with *Pigm* and *Pi2* displayed effective and stable broad-spectrum resistance in multi-location blast nurseries

**Fig. 5:**
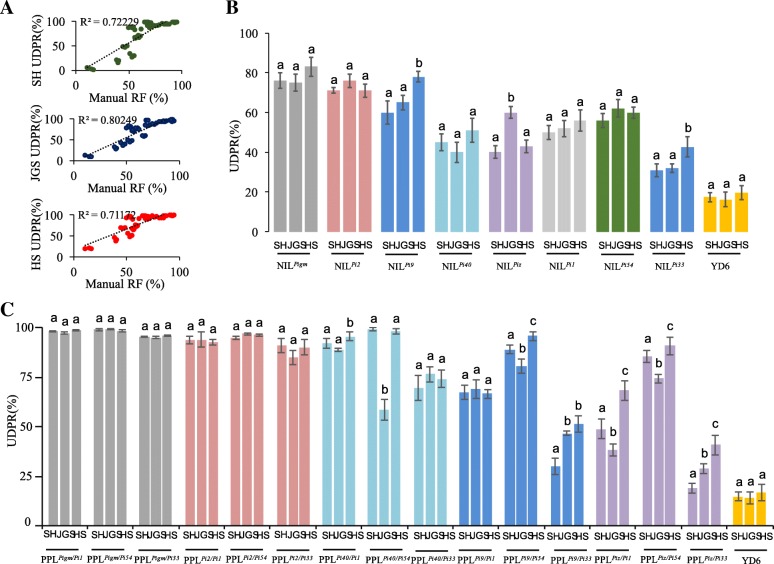
Resistance performances of PPLs in the three blast nurseries. **a** Relationship between HPP of PPLs at three disease nurseries and the RF of PPLs in artificial inoculation evaluation; **b** Resistance variation of NILs among different disease nurseries; **c** Resistance variation of PPLs among different disease nurseries. SH: Shanghang; JGS: Jinggangshan; HS: Huangshan; Different lower-case letters indicate significant differences at the *P* < 0.001 level by one-way ANOVA

To characterize the disease resistance of PPLs under natural conditions with high blast disease pressure, field assays were performed under natural conditions in Shanghang in Zhejiang province, Jinggangshan in Jiangxi province and Huangshan in Anhui province. In the ripe stage (30 days after heading), panicle blast evaluation was represented by healthy panicle proportion (HPP), defined as HPP = (total panicles inoculated - diseased panicles/ total panicles inoculated) × 100%. The recurrent parent YD6 was found to be highly susceptible at these three locations, indicated that these locations possess suitable field conditions for blast disease development and are ideal nurseries. The results showed that the natural evaluation results were consistent with the artificial inoculation identification results, and the determination coefficient (R^2^) between natural evaluation and artificial inoculation identification in Shanghang, Jinggangshan and Huangshan was 0.7223, 0.8025 and 0.7117, respectively (Fig. 5a). From Fig. 5b result, we found that the NIL^*Pigm*^ and NIL^*Pi2*^ showed minimum resistance fluctuation and displayed broader resistance under Shanghang, Huangshan and Jianggangshan test sites. Moreover, PPL^*Pigm/Pi1*^, PPL^*Pigm/Pi54*^, PPL^*Pigm/Pi33*^, PPL^*Pi2/Pi1*^, PPL^*Pi2/Pi54*^ and PPL^*Pi2/Pi33*^ also showed minimum resistance fluctuation and displayed effective and stable broad-spectrum resistance under three test sites (Fig. 5c). Except for PPL^*Pi2/Pi33*^, the HPP of other five PPLs were ranging from 93.05% to 99.65% with resistance scores of 1 to 3. Besides, there were great differences in the panicle blast resistance of PPLs (PPL^*Pi40/Pi1*^, PPL^*Pi40/Pi54*^, PPL^*Pi40/Pi33*^, PPL^*Pi9/Pi1*^, PPL^*Pi9/Pi54*^, PPL^*Pi9/Pi33*^, PPL^*Piz/Pi1*^, PPL^*Piz/Pi54*^ and PPL^*Piz/Pi33*^) at different test sites, such as the HPP of PPL^*Pi40/Pi54*^ was 99.35% and 98.00% and displayed *R* level with resistance scores of 1 in Shanghang and Huangshan, respectively. However, the HPP of PPL^*Pi40/Pi54*^ was only 59.30% and showed S level with resistance scores of 7 in Jinggangshan, indicated that the gene combination of *Pi40*/*Pi54* showed a certain degree of specific compatibility to rice blast fungus populations in Jinggangshan (Fig. 5b, Table 1).

**Table 1:** Resistant performance of panicle blast for PPLs and the recurrent parent at three hotspot locations during the summer season in 2017

Genotypes	Shanghang		Jinggangshan		Huangshan	
	HPP (%)	Disease score	HPP (%)	Disease score	HPP (%)	Disease score
PPLPigm/Pi1PPLPigm/Pi1	98.85 ± 1.05	1	96.00 ± 1.05	1	98.90 ± 0.70	1
PPLPigm/Pi54PPLPigm/Pi54	99.65 ± 0.35	1	99.25 ± 0.25	1	98.20 ± 1.05	1
PPLPigm/Pi33PPLPigm/Pi33	95.20 ± 1.28	1	95.75 ± 2.72	1	98.15 ± 0.61	1
PPLPi2/Pi1PPLPi2/Pi1	95.90 ± 1.05	1	94.40 ± 1.58	3	93.05 ± 3.07	3
PPLPi2/Pi54PPLPi2/Pi54	96.70 ± 0.32	1	96.70 ± 3.69	1	96.35 ± 2.55	1
PPLPi2/Pi33PPLPi2/Pi33	89.45 ± 1.35	5	85.05 ± 4.65	5	90.30 ± 3.68	3
PPLPi40/Pi1PPLPi40/Pi1	92.95 ± 1.47	3	89.90 ± 2.81	5	95.45 ± 2.02	1
PPLPi40/Pi54PPLPi40/Pi54	99.35 ± 0.45	1	59.30 ± 5.09	7	98.00 ± 1.81	1
PPLPi40/Pi33PPLPi40/Pi33	70.00 ± 4.25	7	77.10 ± 6.32	5	74.05 ± 2.73	7
PPLPi9/Pi1PPLPi9/Pi1	69.05 ± 6.41	7	68.80 ± 4.04	7	66.75 ± 6.43	7
PPLPi9/Pi54PPLPi9/Pi54	89.05 ± 3.95	5	81.00 ± 5.79	5	95.85 ± 0.79	1
PPLPi9/Pi33PPLPi9/Pi33	29.95 ± 3.95	9	47.85 ± 6.06	9	51.30 ± 15.63	7
PPLPiz/Pi1PPLPiz/Pi1	50.35 ± 7.11	7	38.85 ± 4.63	9	68.50 ± 2.98	7
PPLPiz/Pi54	85.35 ± 4.83	5	73.70 ± 3.38	7	90.65 ± 2.72	3
PPLPiz/Pi33	19.70 ± 5.45	9	28.95 ± 4.52	9	40.30 ± 5.66	9
YD6	3.25 ± 1.05	9	8.70 ± 1.85	9	20.55 ± 4.65	9

### Agronomic performances of the PPLs

The agronomic traits of PPLs and recurrent parent YD6 were investigated and the results showed that most of the agronomic performance of PPLs, such as PH, PN, TSP, SF, GW and YPP, were similar to those of YD6, only significant variation was observed with respect to PH, DFF, TSP and YPP among the PPL^*Pi2/Pi1*^, PPL^*Pi2/Pi54*^ and PPL^*Pi2/Pi33*^ as compared to YD6 (Table 2). In addition, we found that the yield traits of all the other PPLs were comparable to the recurrent parent, especially the YPP and the other yield component traits of PPL^*Pigm/Pi1*^, PPL^*Pigm/Pi54*^ and PPL^*Pigm/Pi33*^ were at par to YD6, indicating that most of the genetic background that control elite agronomic trait of the recurrent parent were retained in the PPLs after previous agronomic trait selection and whole genome selection.

**Table 2:** Agronomic performance of PPLs and the recurrent parent during the summer season in 2017

Genotypes	PH (cm)	DFF (days)	PN	TSP	GW (g)	SF(%)	YPP (g)
PPLPigm/Pi1	116.25 ± 2.36	101.50 ± 0.50	7.80 ± 0.99	174.40 ± 10.64	30.60 ± 0.50	93.05 ± 1.12	35.15 ± 0.62
PPLPigm/Pi54	117.04 ± 4.60	99.50 ± 1.00	8.45 ± 0.62	174.25 ± 4.84	30.85 ± 0.87	91.65 ± 0.62	35.40 ± 1.74
PPLPigm/Pi33	111.35 ± 5.09	101.00 ± 0.50	8.50 ± 0.75	174.25 ± 10.06	29.95 ± 0.37	93.20 ± 1.74	34.95 ± 0.63
PPLPi40/Pi1	116.05 ± 3.61	100.50 ± 1.24	8.10 ± 0.25	176.50 ± 10.18	29.85 ± 0.62	91.75 ± 1.12	33.85 ± 0.62
PPLPi40/Pi54	114.95 ± 3.35	100.50 ± 1.00	8.05 ± 0.37	173.30 ± 6.21	29.95 ± 0.62	93.05 ± 1.12	34.85 ± 0.87
PPLPi40/Pi33	111.10 ± 5.96	100.50 ± 0.50	8.50 ± 0.50	171.45 ± 5.09	29.70 ± 0.25	91.95 ± 1.61	35.80 ± 0.74
PPLPi9/Pi1	116.00 ± 1.99	99.50 ± 1.24	7.75 ± 0.37	174.90 ± 20.62	29.05 ± 0.62	91.55 ± 2.36	34.80 ± 1.49
PPLPi9/Pi54	117.40 ± 1.99	99.00 ± 2.48	8.20 ± 0.99	172.80 ± 12.67	30.25 ± 0.87	92.25 ± 1.12	34.90 ± 0.50
PPLPi9/Pi33	115.45 ± 1.86	99.00 ± 1.00	7.70 ± 0.99	173.25 ± 7.331	30.15 ± 1.37	92.95 ± 1.12	35.35 ± 1.37
PPLPi2/Pi1	115.10 ± 4.22	94.50 ± 1.50**	8.50 ± 0.99	150.20 ± 5.96**	30.00 ± 0.75	93.50 ± 0.75	32.85 ± 0.87**
PPLPi2/Pi54	113.20 ± 1.49	96.00 ± 1.50*	8.35 ± 0.37	143.40 ± 5.47**	29.05 ± 0.87	92.60 ± 2.24	32.95 ± 0.37**
PPLPi2/Pi33	106.25 ± 2.36**	95.00 ± 1.50**	7.75 ± 0.37	147.35 ± 6.83**	29.00 ± 0.99	91.20 ± 1.49	33.90 ± 0.74*
PPLPiz/Pi1	114.85 ± 0.62	101.50 ± 0.50	8.05 ± 0.62	173.65 ± 3.11	30.00 ± 0.99	93.20 ± 1.49	34.40 ± 0.75
PPLPiz/Pi54	115.00 ± 1.99	98.50 ± 1.00	8.60 ± 0.75	177.75 ± 9.81	29.95 ± 1.12	92.30 ± 2.73	34.10 ± 0.99
PPLPiz/Pi33	113.20 ± 2.24	99.50 ± 0.50	7.90 ± 0.99	176.35 ± 5.34	29.85 ± 1.12	92.25 ± 3.11	35.65 ± 1.37
YD6	114.95 ± 0.62	99.50 ± 0.50	8.05 ± 0.62	175.45 ± 7.57	29.85 ± 0.62	93.10 ± 0.75	35.10 ± 0.25

*: significant differences at *P* < 0.05; **: significant differences at *P* < 0.001

## Discussion

Enhancing the host rice resistance is being considered as the best approach to handle the rice blast disease. Pyramiding of broad-spectrum *R* genes into a rice variety has been proved to be an effective way to control rice blast (Ashkani et al. 2015; Khan et al. 2018). Ellur et al. (2016) introduced *Pi2* and *Pi54* into Basmati rice simultaneously, and found that the PPLs with *Pi2* and *Pi54* were not only effective in northern and eastern parts of India, but also in the southern parts of the country such as Pattambi, Kerala, and Gudalur, Tamil Nadu. Similarly, the rice variety Jefferson with the gene combination of *Pik*/*Piz* has remained resistant since its first application in 1997 (Fjellstrom et al. 2004; McClung et al. 1997). In this study, we constructed a total of 15 PPLs using the NILs with different *R* genes (*Pigm*, *Pi2*, *Pi9*, *Pi40* and *Piz*) from Piz locus as core parents and pyramided with *Pi1*, *Pi33* and *Pi54*, respectively. Seedling blast evaluation results showed that most of PPLs could produce TH, which resulting in the RF of PPLs was significantly higher than that of NILs. The seedling blast resistant frequency of PPL^*Pigm/Pi1*^, PPL^*Pigm/Pi54*^, PPL^*Pigm/Pi33*^, PPL^*Pi9/Pi1*^, PPL^*Pi9/Pi54*^, PPL^*Pi2/Pi1*^, PPL^*Pi2/Pi54*^ and PPL^*Pi2/Pi33*^ were higher than 90%, and their TH was ranging from 0.63% to 9.91%. Similarly, for panicle blast resistance, the RF of PPL^*Pigm/Pi1*^, PPL^*Pigm/Pi54*^, PPL^*Pigm/Pi33*^ and PPL^*Pi2/Pi1*^ were higher than 80%, and their TH was ranging from 10.00% to 30.00%. These results suggested that TH play more important role on enhancing panicle blast resistance than seedling blast resistance. Furtherly, under natural identification at multi-location disease nursery, the PPL^*Pigm/Pi1*^, PPL^*Pigm/Pi54*^, PPL^*Pigm/Pi33*^, PPL^*Pi2/Pi1*^, PPL^*Pi2/Pi54*^ and PPL^*Pi2/Pi33*^ presented minimum resistance fluctuation character, and their agronomic traits were at par with the recurrent parent. Therefore, it indicated that selecting effective *R* genes and pyramiding them in an optimal combination pattern is the vital step in resistance breeding programs. The gene combinations *Pigm/Pi1*, *Pigm/Pi54* and *Pigm/Pi33* exhibited the best resistance level both at seedling and heading stage, which could provide useful genes resource for blast resistance breeding practice. However, *Pi2/Pi1* was excellent in resistance to rice blast after introduced into the background of Yangdao 6, but there may be some genes that control undesirable agronomic traits around the target gene combination, causing so-called linkage drag, which makes it difficult to be applied directly in breeding practice.

Different *R* genes often confer resistance to different isolates, races or biotypes. Combining their resistance broadens the number of races or isolates and increases resistance spectrum (Feechan et al. 2015). In this study, we found that the OE of PPLs is one of the most important factors to improve seedling blast resistance level, and all of the gene combinations with effective resistance had a relatively high OE. For example, the gene combination *Pigm*/*Pi1*, the OE between *Pigm* and *Pi1* was 77.22% after pyramided and caused the RF of PPL^*Pigm/Pi1*^ to be as high as 97.67%, while PPL^*Piz/Pi33*^ with 15.19% of OE showed the lowest seedling blast RF (37.25%). In addition, the CE of PPLs is another important component factor of its broad-pectrum resistance. The larger the CE value, the more the number of isolates of *M. oryzae* collaboratively resisted by the two pyramided *R* genes. Here, we found that the panicle blast resistance was not only related to OE but also related with CE. The OE and CE values could be observed in various degrees to broaden the panicle blast resistance spectrum of PPLs compared with that of monogenic lines. The gene combination *Pigm*/*Pi1* with 80.22% of OE and CE present 94.33% of panicle blast RF, while PPL^*Piz/Pi33*^ with 20.01% of OE and CE showed the lowest seedling blast RF (39.65%). Therefore, choosing *R* gene combination with higher CE value will be useful for improving panicle blast resistance.

Except for OE and CE could improve the resistant spectrum in PPLs, the PIE also could enhance the seedling blast and panicle blast resistance spectrum of PPLs. Although the panicle blast RF of NIL^*Pi2*^ and NIL^*Pi1*^ were 33.33% and 53.33%, respectively, the PPL^*Pi2/Pi1*^ with 30% of PIE value presented 83.33% of panicle blast RF which was higher than that of its parental lines. However, not all gene combinations produce PIE after gene pyramided, some *R* genes combination could also produce NIE on blast resistance (Chen et al. 2018). Such as PPL^*Piz/Pi54*^ produced 33.33% of NIE value, which resulted in the panicle blast RF of PPL^*Piz/Pi54*^ was lower than that of NILs with *Pi54*. Similar result was also reported by Hittalmani et al. (2000), the resistance level of PPLs^*Piz5/Pita*^ was lower than that of the monogenic lines with *Piz5*. Although the interaction effect between combined *R* genes is extremely complex (Chaipanya et al. 2017 and Divya et al. 2014) and the mechanism of NIE produced is still unknown. However, pyramiding broad-spectrum *R* genes, each recognizing a unique set of rice blast fungus population into a single cultivar, is still promising and effective (Ashkani et al. 2015; Pilet-Nayel et al. 2017). Nevertheless, the approaches need careful characterization of the resistance spectrum of the target *R* genes to be used and combining them in an effective pyramiding way against the target pathogen population for crop protection.

## Methods

### Plant materials and pathogens

The recurrent parent *indica* cv. Yangdao 6 (YD6) bred by Lixiahe Agricultural Research Institute of Jiangsu Province, China, and was the two-line restorer line with the largest application area in China. At the same time, as the representative of Chinese *indica* rice, YD6 was the first one selected for genome sequencing research (Yu et al. 2002). The eight near-isogenic lines (NILs) with broad-spectrum *R* resistance genes (*Pigm*, *Pi40*, *Pi2*, *Pi9*, *Piz*, *Pi1*, *Pi54* and *Pi33*) were constructed with YD6 as genetic background.

A set of seven Chinese differential rice cultivars, Tetep, Zhenglong 13, Sifeng 43, Dongnong 363, Kanto 51, Hejiang 18, and Lijangxintuanheigu (LTH) were used to study pathogenicity and subgroups of isolates of *M. oryzae* at the seedling stage. A total of 158 isolates were collected and obtained from the diseased panicles from different parts of the infected fields in Hainan (HN), Guangdong, Guangxi, Hunan, Hubei, Jiangsu, Zhejiang, Anhui, Jiangxi and Sichuan provinces in 2010–2016 (Additional file 3: Table S2). Single spore isolation, strain cultivation, and inoculum preparation were conducted following the procedure reported by Puri et al. (2009).

## Molecular marker assay

### DNA isolation and PCR conditions

Three-weeks-old rice leaves were frozen in liquid nitrogen and stored at − 80 °C until DNA extraction. Genomic DNA was extracted using the rapid extraction method of TPS (Lu and Zheng 1992), PCR amplification was carried out in a 20 μL reaction mixture containing 2.0 μL MgCl_2_ (25 mmol L-1), 2.0 μL 10 × PCR buffer, 1.5 μl of each primer (10 μmol L − 1), 0.4 μL dNTP (10 mmol L-1), 50 ng DNA template, 0.2 μL Taq polymerase enzyme (5 U μL-1) and 11.9 μL ddH_2_O. The PCR program were conducted following the standard protocol (Chen et al. 1997), included pre-denaturation for 5 min at 94 °C, followed by 35 cycles of 45 s at 94 °C 45 s at the annealing temperature indicated in Additional file 4: Table S3, 1 min at 72 °C, and a final extension 72 °C for 10 min. The amplification products were visualized on 8% denaturing polyacrylamide gel or 4% agarose gel based on their relative fragment size.

### Foreground selection by molecular markers

The plants were analyzed to confirm the presence of target genes using gene based/linked markers. Foreground selection for the gene Pi54 was conducted using the gene-based markers PI54–1. The selection for the genes *Pigm*, *Pi9*, *Pi40*, *Pi2*, *Piz*, *Pi1*, and *Pi33* was carried out using specific gene-linked markers ZJ58.7, RM3330, ZJ58.7, AP22, AP5413, RM224 and RM72, respectively, as mentioned in the Additional file 4: Table S3.

### GBS background analysis

Genomic DNA was extracted from 100 mg of leaf tissue using DNAsecure Plant kit reagents following the manufacturer’s protocol (Qiagen, USA). The quality of extracted genomic DNA was measured using BioPhotometer plus (Eppendorf, Germany). Genomic DNA was digested with restriction enzymes BamHI and MspI and sequencing libraries were prepared by ligating the digested DNA to unique nucleotide adapters (barcodes) followed by standard PCR. Sequencing was performed using Illumina HiSeq2000 Sequencer (Illumina, USA) (Poland et al. 2012). The raw Illumina DNA sequence data (FASTQ file) were processed through the GBS analysis pipeline in TASSEL v3.0 software (Bradbury et al. 2007). The raw reads were sorted according to indices, and the high-quality SNPs between parents were called by alignment with Nipponbare reference genome MSU release 7 (Kawahara et al. 2013) using BWA package (Lai et al. 2010; Li and Durbin 2009) and Genome Analysis Toolkit (GATK) (McKenna et al. 2010).

## Evaluation for blast resistance

### Evaluation for blast resistance by artificial inoculation

Eight NILs, fifteen PPLs and seven Chinese differential rice cultivars were screened for blast resistance under artificial conditions using a set of 158 *M. oryzae* isolates. Ten plants of each tested materials were grown in a plastic tray filled with sieved garden soil in greenhouse maintained at 27 to 30 °C till three leaf emergences. Three replicates of all tested materials were included in the inoculations with the recurrent parent YD6 and the standard susceptible check LTH as the susceptible control. Three-week old rice seedlings were inoculated with 40 mL of an *M. oryzae* conidial suspension (5 × 10^4^ conidia/mL) with 0.02% Tween 20 using a hand atomizer (100 kPa) connected to an air compressor. Inoculated plants were incubated for 24 h in the dark in growth chambers maintained at 26 °C. Plants were transferred to the greenhouse post-inoculation under a 12-h light/12-h dark photocycle at 90% relative humidity by intermittent spraying with water. Blast disease score was recorded after seven days according to the standard procedures (Mackill and Bonman 1992), where lines with scores of 0 to 2 were considered resistant (R) and 3 to 5 were considered to be susceptible (S). The blast resistance of each NIL and PPL were identified by three replications.

The NILs, PPLs and the recurrent parent YD6 were screened resistance to panicle blast under natural conditions. A set of 30 isolates were selected from the set of 158 blast isolates for panicle blast resistance screen assays. Selection criteria were based on the virulence patterns in seven Chinese differential rice cultivars as described by Wu et al. (2016). 120 plants of each experimental material were transplanted in the paddy field. Each plot contains 10 rows and 12 plants per row with row spacing 13.3 cm × 25 cm. A completely randomized block design (RCBD) was used with three replications. Individual rice tillers were inoculated at the booting stage (the beginning of panicle initiation), by injecting 1 mL of an *M. oryzae* conidial suspension (5 × 10^4^ conidia/mL) into the panicle of each plant between the second and ninth rows. A total of 10 rice tillers were inoculated with each *M. oryzae* isolate. In ripe stage, the panicle blast evaluation was based on incidence rates of panicle blast symptoms and the standard reference was described by Puri (2009).

### Multi-location disease resistance evaluations in the blast nurseries

The NILs and PPLs were screened for their reaction to blast under Uniform Blast Nursery (UBN) at three hot spot locations viz., Shanghang in Zhengjiang province, Jinggangshan in Jiangxi province and Huangshan in Anhui province, where possess suitable field conditions for blast disease development. Each plot had five rows and 12 plants per row with row spacing 13.3 cm × 25 cm, and a completely randomized block design was used with three replications. The susceptible checks were planted as a spreader in 2 rows at both sides of each block to maximize the disease incision. The water layer of field was about 10 cm and no fungicide was used. The disease score was recorded on the 0–5 standard evaluation scale of IRRI (2002) with slightly modified as follows: lines with 0 score were considered as highly resistant (HR) and there were no diseased plant in the plot, 1 score was resistant (R) and the health panicle proportion (HPP) in the plot was higher than 95.0%, 2 was moderately resistant (MR) and the HPP in the plot was ranging from 90.1% to 95.0%, 3 was moderately susceptible (MS) and the HPP in the plot was ranging from 75.1% to 90%, 4 was susceptible (S) and the HPP in the plot was ranging from 50.1% to 75%, where 5 were highly susceptible (HS) and the HPP in the plot was ranging from 0% to 50%.

### Evaluation of PPLs for agronomic performance

Evaluation of agronomic traits under natural field condition was conducted in the field at Lixiahe Agricultural Research Institute of Jiangsu Province, China (32°38’ N 119°43′ E). Performance of the PPLs and recurrent parent YD6 were evaluated during the summer of 2017. Each line was planted in a Plot of seven rows with 12 plants per row as described above, and a RCBD with two replications. Normal water and fertilizer management, disease and pest control were conducted. Five plants in the middle of each plot were taken randomly for measurements of days to 50% flowering (DFF), plant height (PH), panicle number per plant (PN), total spikelets per plant (TSP), spikelet fertility (SF), 1000 grain weight (GW), and yield per plant (YPP), according to the standard evaluation system for rice (IRRI 2002).

### Data analysis

The seedling blast and panicle blast resistance was represented by resistance frequency (RF), defined as RF = (number of incompatible *M. oryzae* isolates/total number of *M. oryzae* isolates inoculated) × 100% (Wu et al. 2016). The transgressive heterosis number is calculated by using this formula: Transgressive heterosis (TH) = RF^PPL^ – RF^NIL^. RF^PPL^ refers to the resistance frequency of PPL; RF^NIL^ represents the resistance frequency of the NIL with the highest resistance frequency. The relationship between different interaction effect and RF was analyzed using a multiple stepwise regression model, which is an available option in Matlab (V.7.0) software (Xu et al. 2012). The interaction effects that were significantly correlated with RF were subjected to clustering by principal component analysis (PCA) in SPSS (V.21) software.

## Additional files

Additional file 1: **Table S1.** Information of insert fragments from donor to receptor for each of the PPLs. (XLSX 11 kb)

Additional file 2: **Figure S1.** Comparing of resistant spectrum between PPLs and NILs. A Seedling blast stage; B Panicle blast stage. The number in this picture is total amount of blast isolate resistant to PPLs or NILs. (PDF 70 kb)

Additional file 3: **Table S2.** Information of blast populations for pathogenicity assays. (XLSX 19 kb)

Additional file 4: **Table S3.** Detail information of molecular markers tightly linked to different resistant genes. (DOCX 16 kb)
