# Supplementary figures and images for: Comprehensive evaluation of resistance effects of pyramiding lines with different broad-spectrum resistance genes against Magnaporthe oryzae in rice (Oryza sativa L.)
Source: Rice (N Y). 2019 Mar 1;12:11. doi: 10.1186/s12284-019-0264-3 (PMC6397272; doi:10.1186/s12284-019-0264-3)

## Seedling Blast

## Panicle Blast

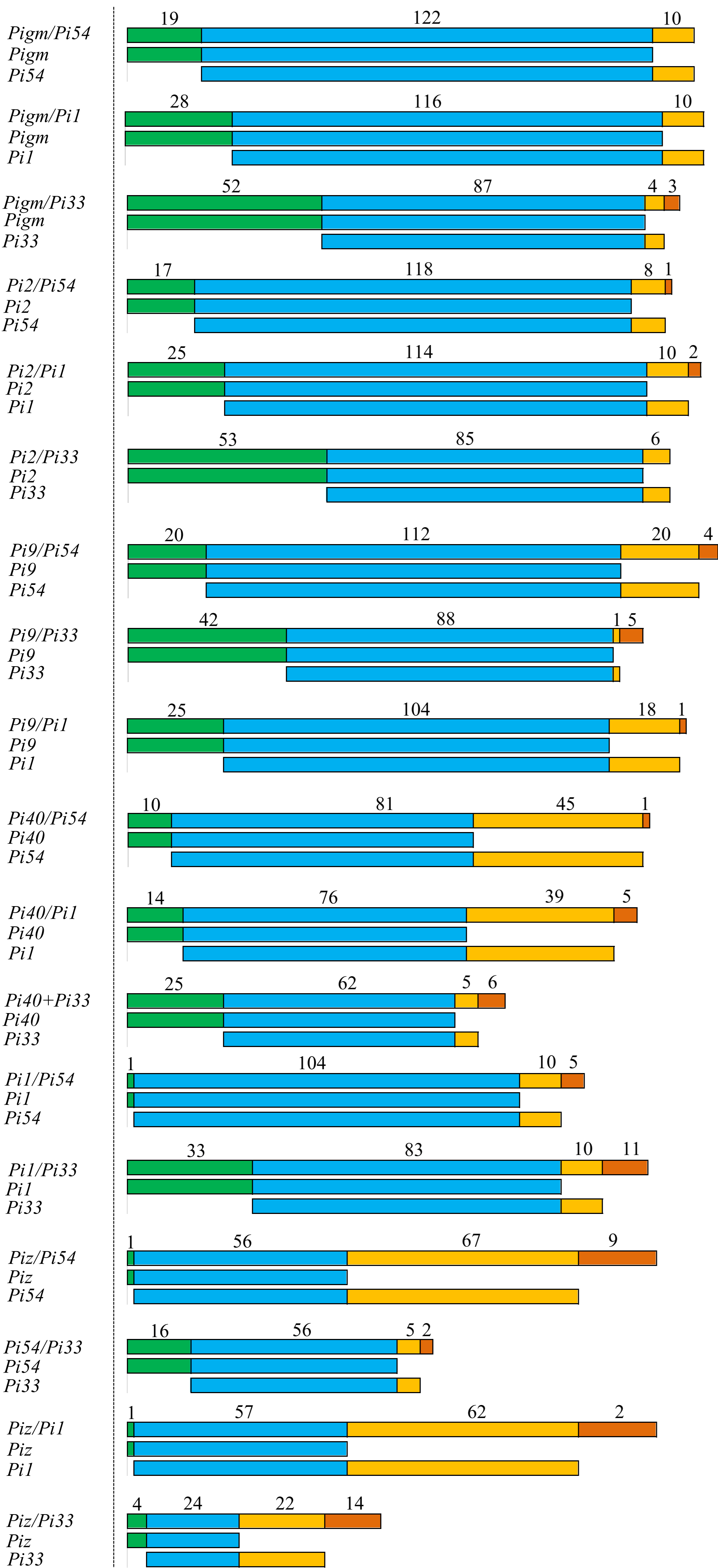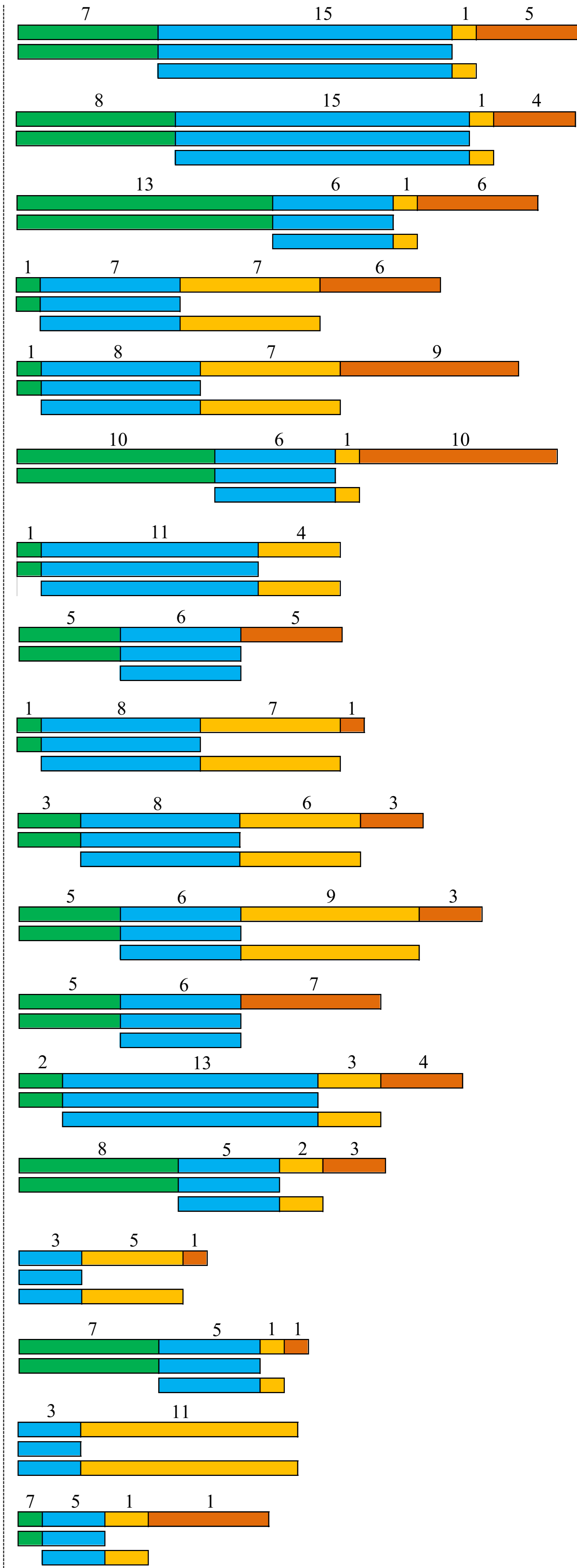

CE OE PIE

Supplement: Supplementary file 2 — Figure S1. Comparing of resistant spectrum between PPLs and NILs. A Seedling blast stage; B Panicle blast stage. The number in this picture is total amount of blast isolate resistant to PPLs or NILs. (PDF 70 kb) [file 12284_2019_264_MOESM2_ESM.pdf]
