# Supplementary material for: Comprehensive evaluation of resistance effects of pyramiding lines with different broad-spectrum resistance genes against Magnaporthe oryzae in rice (Oryza sativa L.)
Source: Rice (N Y). 2019 Mar 1;12:11. doi: 10.1186/s12284-019-0264-3 (PMC6397272; doi:10.1186/s12284-019-0264-3)
Supplement: Supplementary file 4 — Table S3. Detail information of molecular markers tightly linked to different resistant genes. (DOCX 16 kb) [file 12284_2019_264_MOESM4_ESM.docx]

Table S3 Detail information of molecular markers tightly linked to different resistant genes

| Genes | Marker | Primer sequence (5′–3′) | Annealing temperature (℃) | Expected size (bp) |
| --- | --- | --- | --- | --- |
| *Pi2* | AP22 | F: GTGCATGAGTCCAGCTCAAA  R: GTGTACTCCCATGGCTGCTC | 58 | 143 |
| *Pi9* | RM3330 | F: CGTTCGAGCAGAACCATCTACC  R: CCTCTTCCGCTCCACTCTCC | 58 | 170 |
| *Pi40* | ZJ58.7 | F: ACTTGCTGGGAGAAGGATT  R: AGTTCGTACTTTTCAGGCT | 55 | 236 |
| *Pigm* | ZJ58.7 | F: ACTTGCTGGGAGAAGGATT  R: AGTTCGTACTTTTCAGGCT | 55 | 236 |
| *Piz* | AP5413 | F: GAAAGTGGGTTAAGGGACAC  R: GAGTCTGTCAAGATTAAGATTCAG | 55 | 334 |
| *Pi1* | RM224 | F: ATCGATCGATCTTCACGAGG  R: TGCTATAAAAGGCATTCAAA | 55 | 163 |
| *Pi33* | RM72 | F: CCGGCGATAAAACAATGAG  R: GCATCGGTCCTAACTAAGGG | 55 | 240 |
| *Pi54* | PI54-1 | F: CAATCTCCAAAGTTTTCAGG  R: GCTTCAATCACTGCTAGACC | 55 | 216 |
